# DISH Phagia!

**DOI:** 10.4103/1319-3767.56096

**Published:** 2009-10

**Authors:** P. V. Santosh Rai

**Affiliations:** Department of Radiodiagnosis, KMC Hospital, Attavar, Mangalore - 575 001, Karnataka, India. E-mail: radiorai@gmail.com

**Figure 1:**
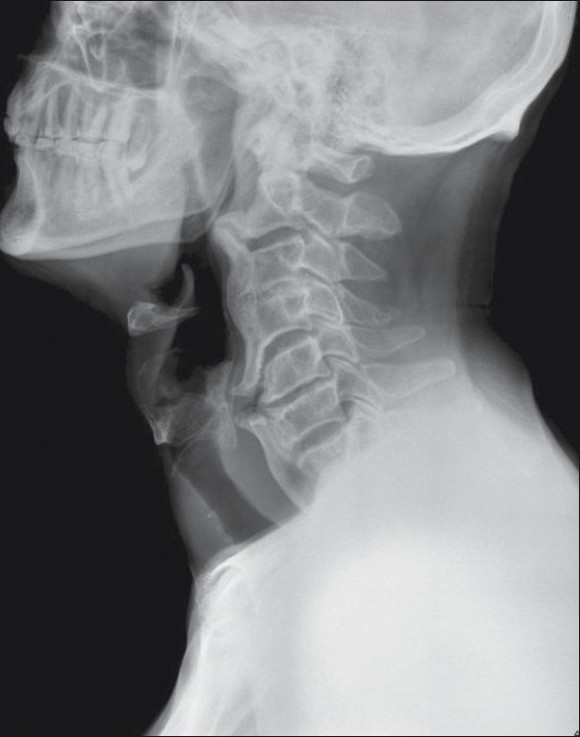
The lateral cervical spine radiograph of this young patient with dysphagia shows flowing ossification along the anterior aspect of the cervical vertebrae, with relative preservation of the disc spaces

Sir,

A young adult was referred to us for evaluation of his difficulty in swallowing. Before we proceeded with his barium swallow, a lateral radiograph of his cervical spine was performed, which showed characteristic flowing ossification along the anterior aspect of the cervical vertebrae, with relative preservation of the disc spaces [Figure 1]. The features were characteristic of diffuse idiopathic skeletal hyperostosis (DISH) or Forestier's disease. The age of presentation in this case was unusual, which warranted reporting in literature.

Dysphagia due to cervical osteophytes is uncommon. However, DISH with cervical involvement, which causes dysphagia is even rarer.[1] Dyspnea with or without dysphagia caused by hypertrophic anterior cervical osteophytes is also an uncommon entity.[2] The diagnosis can be made by plain cervical X-ray films, a barium swallowing esophagogram and/or a CT scan of the neck.

DISH is a condition characterized by calcification and ossification of soft tissues, mainly ligaments and enthesis.[3] Dysphagia is the most common complaint, and stridor secondary to osteophyte compression has rarely been documented. The osteophytes may cause symptoms by mechanical compression or by inducting inflammatory reaction. When an upper segment of the C-spine is involved, particularly C3-C4 level, the larynx may be affected. This could be a result of hoarseness, stridor, laryngeal stenosis and obstruction. Sometimes vocal fold paralysis may result from injury to the recurrent laryngeal nerve. Treatment of breathing problems first required stabilization of the airway with tracheostomy. Subsequently, osteophysectomy is performed, which generally provides relief to patients from symptoms.

Three criteria are required to diagnose spinal involvement in DISH:

1. Flowing calcification and ossification along the anterolateral aspect of at least four contiguous vertebral bodies with or without pointed excrescences.
2. Relative preservation of intervertebral disc height and absence of extensive changes typical of degenerative disc disease.
3. Absence of bone ankylosis of the apophyseal joints or of erosion, sclerosis, or intra-articular osseous fusion of the sacroiliac joints.

In conclusion, DISH should be considered an important, although rare, cause of dysphagia among older adults[4]. An awareness of such a cause of dysphagia is required. In our case, the age of presentation was more unusual.
